# Chikungunya virus adaptation to *Aedes albopictus *mosquitoes does not correlate with acquisition of cholesterol dependence or decreased pH threshold for fusion reaction

**DOI:** 10.1186/1743-422X-8-376

**Published:** 2011-07-29

**Authors:** Konstantin A Tsetsarkin, Charles E McGee, Stephen Higgs

**Affiliations:** 1 Department of Pathology, University of Texas Medical Branch, Galveston, Texas, USA; 2 Carolina Vaccine Institute, University of North Carolina, Chapel Hill, North Carolina, USA

**Keywords:** Chikungunya virus, *Aedes albopictus*, cholesterol, pH threshold of fusion

## Abstract

**Background:**

Chikungunya virus (CHIKV) is a mosquito transmitted alphavirus that recently caused several large scale outbreaks/epidemics of arthritic disease in tropics of Africa, Indian Ocean basin and South-East Asia. This re-emergence event was facilitated by genetic adaptation (E1-A226V substitution) of CHIKV to a newly significant mosquito vector for this virus; *Aedes albopictus*. However, the molecular mechanism explaining the positive effect of the E1-A226V mutation on CHIKV fitness in this vector remains largely unknown. Previously we demonstrated that the E1-A226V substitution is also associated with attenuated CHIKV growth in cells depleted by cholesterol.

**Methods:**

In this study, using a panel of CHIKV clones that varies in sensitivity to cholesterol, we investigated the possible relationship between cholesterol dependence and *Ae. albopictus *infectivity.

**Results:**

We demonstrated that there is no clear mechanistic correlation between these two phenotypes. We also showed that the E1-A226V mutation increases the pH dependence of the CHIKV fusion reaction; however, subsequent genetic analysis failed to support an association between CHIKV dependency on lower pH, and mosquito infectivity phenotypes.

**Conclusion:**

the E1-A226V mutation probably acts at different steps of the CHIKV life cycle, affecting multiple functions of the virus.

## Introduction

Chikungunya virus (CHIKV), an arthritogenic alphavirus, has received global attention due to the series of recent large-scale outbreaks in different parts of the world including Africa, Indian Ocean Islands, India and countries of South-East Asia [1,2]. The virus was also introduced with viremic travelers into Europe where it caused, for the first time, autonomously transmitted outbreaks in 2007 [3] and 2010 [4]. The global expansion of CHIKV was at least partially attributed to the viral adaptation to a new mosquito vector *Aedes (Ae.) albopictus*, which facilitated CHIKV transmission in the regions that lack typical vector *Ae. aegypti *[5-10]. Phylogenetic studies have identified a specific mutation (E1-A226V) in the E1 glycoprotein of CHIKV strains circulating in the areas where *Ae. albopictus *serves as primary virus vector [6,8,9,11-13]. Additionally, laboratory studies confirmed that the E1-A226V substitution leads to increased CHIKV infectivity and is associated with more efficient dissemination and transmission by *Ae. albopictus *[14,15]. We also demonstrated that midgut epithelial cells of *Ae. albopictus *appear to be the primary target cells where this mutation has the most profound effect [16]. However, the molecular mechanism underlying the positive effect of E1-A226V on CHIKV infectivity for midgut cells of *Ae. albopictus *remains largely unknown.

The envelope of CHIKV consists of 240 copies of membrane-embedded E2-E1 heterodimers, which surround the nucleocapsid core. Cellular entry is mediated by the interaction of E2 glycoprotein with an unidentified receptor, followed by endocytosis of the virion-receptor complex into endosomal compartments. Acidification of endosomes subsequently triggers a cascade of events that culminates in E1 activation and viral fusion reaction [17-19]. Since the mutation responsible for adaptation of CHIKV to *Ae. albopictus *occurs in the E1 protein; a receptor-mediated explanation of its effect on viral fitness in this mosquito seems implausible, suggesting that this mutation acts at steps in the virus life cycle following receptor-mediated endocytosis.

Previously it has been shown that mutations at the same position of the E1 protein (E1-226) of other alphaviruses; Semliki Forest virus (SFV) and Sindbis virus (SINV), can modulate the cholesterol requirements for viral entry into, and exit from, cells [20-23]. Thus, the single amino acid substitution E1-P226S in SFV and triple substitution at (E1-A226S, E1-K227G, E1-N228M) in SINV was sufficient to significantly increase virus production in, virus infectivity to, and virus exit from cholesterol-depleted *Ae albopictus *(C6/36) cells [22,23]. Position E1-226 is located at the tip of the ij loop of the E1 protein of alphaviruses [24,25] and it was hypothesized that the presence or absence of cholesterol in target membranes is detected by residues in this loop, which influences the kinetics of E1 conformational changes during fusion [26]. Interestingly, in a study using Venezuelan equine encephalitis virus (VEEV)-enveloped pseudo-typed murine leukemia virus, it was shown that the entry of VEEV into cells is resistant to cholesterol depletion of the mammalian cells [27], suggesting that cholesterol dependence is not absolute and probably depends on particular virus/host species relationships.

Previously, we demonstrated that the E1-A226V mutation that confers increased fitness of CHIKV in *Ae. albopictus *is also associated with CHIKV dependence for cholesterol for growth in mosquito derived cell lines. Thus, the viruses that have alanine at E1-226 grew to 4-5 Log_10_TCID_50_/mL higher titers in cholesterol-depleted C6/36 cells as compared to viruses that have valine at this position [15]. However, no difference in growth kinetics between these viruses was detected in standard C6/36 cells, suggesting that dependence for cholesterol might be directly connected to the mosquito infectivity phenotype.

In this study, to investigate possible relationship between cholesterol dependence of CHIKV and *Ae. albopictus *infectivity, we generated a panel of CHIKV clones that varies in sensitivity to cholesterol and evaluated their relative infectivites for *Ae. albopictus *mosquitoes.

## Materials and methods

### Plasmids and cells

The plasmids encoding full-length infectious clones of the LR2006 OPY1 strain pCHIKV-LR i.c. (GenBank accession number EU224268), GFP-expressing full length clone pLR-GFP-226V (pCHIKV-LR 5'GFP, GenBank accession number EU224269), pLR-ApaI-226V and pLR-226A have been previously described [15,28]. A series of point mutations at E1-226 (S, T, G, I, P, F, M, H, L) was introduced into pCHIKV-LR i.c. using standard molecular biology methods that resulted in clones designated as pLR-226X, where X corresponds to one of residues to be introduced. To introduce S, T, G, I, P, F, M, H, and L residues individually at E1-226 in the background of eGFP-expressing CHIKV pLR-GFP-226V [15], the DNA fragment of 4709 nt. was generated by digestion of pLR-GFP-226V with *Age*I and *Xho*I. This fragment was cloned into each of pLR-226X plasmids by *Age*I and *Xho*I sites. The resultant plasmids were designated pLR-GFP-226X. Presence of the eGFP gene in the plasmid was confirmed by restriction digestion analysis.

A plasmid encoding the pCHIKV-LR i.c. containing mutations identified in the cholesterol-independent plaque purified clone of LR-ApaI-226V (Clone#1) [see below; adaptation of LR-ApaI-226V to cholesterol-depleted C6/36 cells] was generated by cloning of two cDNA fragments into pCHIKV-LR 3**'**GFP [28]. Fragment 1 (1386 nt) was amplified based on RNA isolated form Clone#1 virus using CHIKV specific primers, and digested with *Xho*I and *Kpn*I restrictases. Fragment 2 (961 nt) was generated by digestion of pLR-GFP-226V with *Kpn*I and *Pac*I. Fragments 1 and 2 were simultaneously ligated and cloned by *Xho*I and *Pac*I sites into pCHIKV-LR 3'GFP. The resultant plasmid was named pLR-Cl#1. To introduce a single point mutation, C10660A, into pLR-Cl#1, two DNA fragments of 1082 nt. and 1249 nt. were generated by digestion of pLR-Cl#1 with *Xho*I and *EcoR*V and digestion of pLR-GFP-226V with *EcoR*V and *Pac*I restrictases, respectively. Both DNA fragments were cloned simultaneously into pCHIKV-LR 3**'**GFP by *Xho*I and *Pac*I sites. The eGFP expressing version of this plasmid was generated using the same strategy as was described for pLR-GFP-226X constructs. The resultant clone was designated as pLR-GFP-Cl#1-10660A and sequenced.

The presence of correct nucleotide substitutions leading to the desired amino acid mutations at specific position of E1 gene was confirmed by sequencing analysis. All clones possessed mutations of interest and no additional substitutions were detected. Plasmids were propagated in MC1061 strain of *E.coli *and purified using the QIAprep Spin Miniprep Kit (Qiagen, Valencia, CA) following manufacturer's protocol. Detailed information for all constructs is available from authors upon request.

BHK-21 (Baby Hamsters Kidney) and Vero (African green monkey kidney) cells were maintained at 37°C with 5% CO_2_. BHK-21 were propagated in MEM alpha (Invitrogen, Carlsbad, CA) supplemented with 10% FBS and 1× MEM vitamin solution (Invitrogen, Carlsbad, CA). Vero cells were maintained in MEM (Invitrogen, Carlsbad, CA) supplemented with 5% BGS and 1× MEM non-essential amino acids solution (Invitrogen, Carlsbad, CA). C6/36 cells (*Ae. albopictus*) were maintained at 28°C without CO_2 _in Leibovitz L-15 (L-15) medium supplemented with 10% FBS.

### Rescue of infectious viruses from infectious clones

One microgram of plasmid DNA was linearized with *Not*I restrictase and was used as a template for *in-vitro *transcription reaction using the mMESSAGE mMACHINE kit (Ambion, Austin, TX) according to the manufacturer's protocol. Ten micrograms of *in-vitro *transcribed RNA were electroporated without further purification into 10^7 ^BHK-21 cells using Gene Pulser Xcell electroporation system (Biorad, Hercules, CA) and preset BHK-21 conditions as described previously [29]. Electroporated cells were maintained in 75 cm^2 ^tissue culture flasks at 37°C in 15 mL of L-15 medium. Cell culture supernatants were collected at 24 and 48 h post-infection (hpi), and stored at -80°C. Viral titers were determined by titration of the infectious samples on Vero cells in 96-well plate using standard techniques [29]. Titers were expressed as tissue culture infectious dose 50% endpoint per milliliter of media (Log_10_TCID_50_/mL) [29].

Specific infectivity of electroporated RNA was determined as previously described [28]. Briefly, 1 × 10^5 ^of electroplated BHK-21 cells were ten-fold diluted and transferred onto sub-confluent monolayers (1 × 10^6 ^cells/well) of uninfected Vero cells in six-well plates [28]. Cells were incubated for 2 h to allow attachment to the plate, and the media was replaced with 2 mL of 0.5% agarose in MEM supplemented with 3.3% FBS. Plates were incubated for 48-96 h until visible plaques developed. The results (specific infectivity values) were expressed as pfu/1 μg of electroporated RNA [Additional file 1, Table S1 and Table S2].

### *In vitro *growth of CHIKV in standard and cholesterol-depleted C6/36 cells

To investigate if the mutations in the E1 protein influenced cholesterol dependence of CHIKV, cholesterol-depleted C6/36 cells were generated by five passages in MEM alpha medium containing 10% FBS treated with 2% CAB-O-Sil (Acros Organics) for 12 h at room temperature as previously described [30]. CAB-O-Sil is a hydrated colloidal silica that adsorbs lipoproteins/cholesterol and allows removal of cholesterol from biological solutions. CHIKV growth curves were determined by infecting cholesterol-depleted and normal C6/36 cells duplicates in 25 cm^2 ^flasks at multiplicities of infection (MOI) of 0.1 and 1.0, respectively, by rocking for 1 h at 25°C. The cells were washed three times with medium and 5 mL of fresh L-15 or MEM alpha medium supplied with 10% of standard or CAB-O-Sil treated FBS was added to the flask. Five hundred microliters of medium was collected from each flask at the indicated times post-infection, and medium volume was adjusted by adding 0.5 mL of appropriate medium. Viral titers were determined by titration of cell culture samples on Vero cells.

### C6/36 cells infectivity assay

Primary infections of cholesterol-depleted C6/36 cells with CHIKV containing specific mutations in the E1 glycoprotein were quantified using an infectious centers assay. Confluent monolayers of cholesterol-depleted C6/36 cells in 96 well plates were infected with 10 μL of serial ten-fold dilutions of eGFP expressing CHIK viruses, for 1 h at room temperature. The virus dilutions were made in MEM alpha medium containing 10% FBS treated with 2% CAB-O-Sil. Following infection, 100 μL of MEM alpha medium containing 10% cholesterol-depleted FBS and 0.8% of carboxy methyl cellulose (which prevents spread of viruses beyond the initial focus of infection) were added to each well. Plates were incubated for 20 h at 30°C. Experiments were performed in triplicates, and eGFP expressing foci were counted and results were normalized to 10^6 ^infectious centers/mL for standard C6/36 cells.

### Adaptation of LR-ApaI-226V to cholesterol-depleted C6/36 cells

LR-ApaI-226V virus was passed four times in cholesterol-depleted C6/36 cells maintained in L-15 medium. At first passage, a confluent monolayer of cholesterol-depleted C6/36 in 25 cm^2 ^tissue culture flask was infected with LR-ApaI-226V at an MOI of 0.1. At 3 days post infection (dpi), 1 mL of tissue culture supernatant was used to infect new cholesterol-depleted C6/36 in 25 cm^2 ^tissue culture flasks. After the fourth passage in cholesterol-depleted C6/36 cells, tissue culture supernatant was harvested at 2 dpi and stored at -80°C until needed. To obtain the plaque-purified clones, supernatant of the passage four was titrated by plaque assay on Vero cells as previously described [31]. At 2 dpi, ten individual plaques below the agarose were pierced with a 200 μL tip and used for infection of standard C6/36 cells in L-15 medium in one well of a 12 well plate. At 2 dpi culture supernatants were harvested, titrated and stored at -80°C until needed. The 9500-11000 nt. genome region of the plaque-purified clones was sequenced.

### Virus competition assay in cells treated with NH_4_Cl

BHK-21 or C6/36 cells in 12 well plates were maintained in L-15 medium supplied with 10% FBS. Thirty minutes prior to infection, cell culture media was replaced with 0.5 mL of the L-15 media containing various amounts of NH_4_Cl (endosomal acidification inhibitor) as indicated (see results). After a 30 min incubation at room temperature, 50 μL of a 1:1 mixture of LR-ApaI-226V and LR-226A virus (10^7^pfu/mL) was added to each well and infection was allowed to continue for 1 h at room temperature. Cells were washed once with 0.8 mL of L-15 media containing NH_4_Cl, then 0.8 mL of fresh L-15 medium containing various amounts of NH_4_Cl was added to each well and cells were incubated for 48 h at 37°C for BHK-21 or 28°C for C6/36 cells. Cell culture supernatants were harvested and used for RNA extraction, or titration on Vero cells. Virus RNA was processed as discussed previously [15].

### Cell-cell fusion assay

For comparative studies of the pH threshold of the CHIKV fusion reaction, confluent monolayers of C6/36 cells in 24-well plate were infected (MOI of 0.5-1.0) with eGFP expressing CHIKV containing specific mutations in the E1 glycoprotein. Cells were incubated for 24 h in 0.5 mL of L-15 medium supplemented with 10% FBS to allow cell surface expression of viral glycoproteins. Cells were washed once with 0.5 mL of L-15 and the cell-to-cell fusion reaction was triggered by incubating the cell monolayers for 2 min at 30°C in 1 mL of pre-warmed (30°C) L-15 medium whose pH was previously adjusted to desired values. Fusion reactions were stopped by replacement of the fusion medium with 0.5 mL of standard L-15 medium (pH 7.4). Cells were incubated for 3 h at 28°C to allow polykaryons to develop. Cells were fixed with 3.5% formaldehyde and stained with 1 μg/mL of 4',6-diamidino-2-phenylindole (DAPI) to visualize nuclei. Cells were analyzed by fluorescent microscopy and the fusion index (percent of fusion) was calculated as (1-c/n)x100%, where c is the number of eGFP expressing cells, n is the number of nuclei (n≥70). Experiments were performed in duplicates for each virus.

### Oral infection of mosquitoes

*Ae. albopictus *(Galveston strain) were infected in an Arthropod Containment Level 3 (ACL-3) insectary as described previously [32,33]. To estimate the Oral infectious dose 50% (OID_50_) values, serial ten-fold dilutions of BHK-21 derived viruses were made in L-15 medium followed by mixing equal volume of virus samples with defibrinated sheep blood (Colorado Serum Company, Denver, CO). Blood meals were presented to 4-5 day old female *Ae. albopictus *(100 mosquitoes per virus dilution) using Hemotek feeding apparatus (Discovery Workshops, Accrington, Lancashire, United Kingdom) as described previously [15]. Fully engorged mosquitoes were sorted on ice and placed in carton boxes for maintenance at 16/8 h light/dark photoperiod at 28°C and 80% humidity, with a 10% sucrose solution provided *ad libitum*. Midguts were dissected at 7 dpi and virus-induced eGFP expression in midgut epithelium was analyzed by fluorescence microscopy. CHIKV infection of mosquitoes was detected by observation of CHIKV-expressed eGFP in epithelial cells. The oral infectious dose 50% (OID_50_) values and fiducial limits were calculated using PriProbit (version 1.63) and SAS equivalent method assuming normal function distribution and an "all or nothing" response parameter. For stability assays, 3-4 mosquitoes infected with the highest dilution of the selected viruses were triturated in 1 mL of TRIzol reagent (Invitrogen, Carlsbad, CA) at 7 dpi, and total mosquito and viral RNA was extracted according to the manufacturer's instructions. Viral cDNA was amplified, and the region of interest was directly sequenced using an ABI PRISM model 3100 Genetic Analyzer (Applied Biosystems, Foster City, CA).

## Results

**Figure 1:**
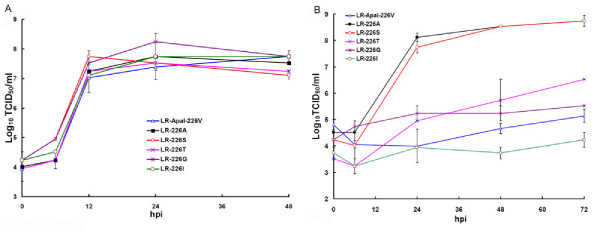
**Effect of S, T, G and I residues at E1-226 on growth of CHIKV in standard (A) and cholesterol-depleted (B) C6/36 cells**. Standard cells were infected at an MOI of 1.0. Cholesterol-depleted cells were infected at an MOI of 0.1. Viral titers are expressed as Log_10_TCID_50_/mL ± standard deviation of 2 independent experiments. hpi - hours post-infection.

**Figure 2:**
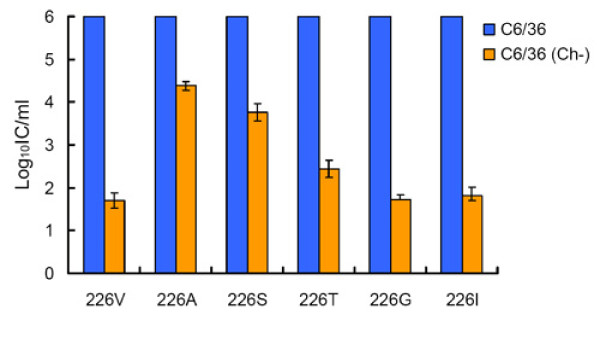
**Effect of S, T, G and I residues at E1-226 on CHIKV infectivity for cholesterol-depleted C6/36 cells**. Standard (blue bars) and cholesterol-depleted (yellow bars) C6/36 cells were infected with serial dilutions of eGFP expressing CHIKV containing indicated residues at E1-226. Results are normalized for 10^6 ^viral infections of standard C6/36 cells. Data indicate an average of three experiments ± standard deviation. IC-infectious center.

To better understand the amino acid requirements at position E1-226 for increased CHIKV midgut infectivity of *Ae. albopictus*, and to understand how it might correlate with cholesterol dependence of CHIKV, a number of CHIKV containing a single amino acid substitutions at E1-226 were constructed and tested in cholesterol-depleted C6/36 cells and in *Ae. albopictus*. Initially, mutations encoding S, T, G, and I residues at E1-226 were introduced into the backbone of non-eGFP expressing pCHIKV-LR i.c. Specific infectivity values were comparable for all of the constructs, indicating that introduction of these residues at E1-226 is not lethal for CHIKV replication in BHK-21 cells (Additional file 1, Table S1). Growth kinetics of LR-226S, LR-226T, LR-226G and LR-226I were determined in standard and cholesterol-depleted C6/36 cells, and compared with growth of LR-ApaI-226V and LR-226A viruses, which have been tested previously [15]. All viruses grew very efficiently in standard C6/36 cells (Figure 1A), however, in cholesterol-depleted cells only LR-226S demonstrated growth kinetics comparable with LR-226A. The growth of all other viruses was significantly attenuated, the peak titers being 3-4 Log_10_TCID_50_/mL lower at 24, 48, and 72 hpi as compared with LR-226A and LR-226S (Figure 1B). To further investigate the effects of S, T, G and I residues at E1-226 on the CHIKV requirement for cholesterol, the mutations encoding these amino acids were introduced into the backbone of eGFP-expressing i.c. of CHIKV (Additional file 1, Table S2), and then specific infectivity of these viruses were determined in standard and cholesterol-depleted C6/36 cells (Figure 2). Relative infectivity of LR-GFP-226A and LR-GFP-226S for cholesterol-depleted C6/36 was 2-2.5 Log_10_IC/mL higher than the infectivity of CHIKV with V, G, or I residues. This is in agreement with the growth kinetics of these viruses in cholesterol-depleted C6/36 cells, indicating that cell infectivity of E1-226V, E1-226G and E1-226I containing virus is much more dependent on the presence of cholesterol than infectivity of E1-226A and E1-226S viruses. Interestingly, the cell infectivity of LR-GFP-226T virus was significantly lower (p < 0.01 Student's t-test) than LR-GFP-226A and LR-GFP-226S viruses, but was significantly higher than that of LR-GFP-226V, LR-GFP-226G and LR-GFP-226I viruses, indicating CHIKV dependence for cholesterol can be expressed as a spectrum of different phenotypes (Figure 2).

To investigate the relationship between CHIKV dependence for cholesterol and CHIKV infectivity for midgut cells of *Ae. albopictus *mosquitoes, serial dilutions of LR-GFP-226S, LR-GFP-226T, LR-GFP-226G and LR-GFP-226I viruses were orally presented to *Ae. albopictus *mosquitoes. CHIKV infectivity was analyzed by the expression of eGFP in the midgut cells at 7 dpi (Table 1). OID_50 _values of LR-GFP-226S, LR-GFP-226T and LR-GFP-226G in *Ae. albopictus *were between 5.35 and 5.94 Log_10_TCID_50_/mL, which were not significantly different from the OID_50 _value of LR-GFP-226A virus (5.45 Log_10_TCID_50_/mL). This indicates that cholesterol dependence of CHIKV does not directly correlate with an increase in midgut infectivity. LR-GFP-226T and LR-GFP-226G both were significantly more cholesterol-dependent as compared to LR-GFP-226A and LR-GFP-226S; however, all four viruses demonstrated almost identical infectivity for *Ae. albopictus *mosquitoes. Infectivity of LR-GFP-226I was almost identical to infectivity of epidemic CHIKV containing the E1-226V residue (p < 0.1). Both valine (V) and isoleucine (I) have additional methyl and ethylene groups in their side chains as compared to alanine or glycine, which are probably required for an increase in CHIKV infectivity for midgut cells of *Ae. albopictus*. The presence of a hydroxyl group in the side chains of serine and threonine apparently has a negative effect on CHIKV infectivity to *Ae. albopictus*.

**Table 1:** Effect of mutations at E1-226 and (E1-66/E1-70) on CHIKV infectivity for *Ae. albopictus *midgut cells

Backbone	E1 226	Exp	N m	OID_50_	FL_95_	p-value
LR-GFP-226A	A	Com	194	5.45	NG	-
LR-GFP-226V	V	Com	261	3.52	NG	< 0.01
LR-GFP-226S	S	1	70	5.94	5.68-6.24	> 0.1
		2	131	5.68	5.45-5.92	> 0.1
LR-GFP-226T	T	1	72	5.35	5.09-5.61	> 0.1
LR-GFP-226G	G	1	107	5.93	5.67-6.19	> 0.1
LR-GFP-226I	I	1	98	3.54	1.94-4.02	< 0.01
LR-GFP-226P	P	1	98	4.78	4.47-5.10	< 0.05
		2	137	4.86	4.43-5.11	< 0.05
LR-GFP-226F	F	1	112	5.62	5.32-5.99	> 0.1
LR-GFP-226M	M	1	117	3.30	2.49-3.68	< 0.01
LR-GFP-226H	H	1	119	4.69	4.44-4.91	< 0.05
		2	99	4.68	4.40-4.93	< 0.05
LR-GFP-226L	L	1	105	3.85	3.57-4.16	< 0.01
LR-GFP-Cl#1-10660A	V	1	80	3.41	3.32-3.50	< 0.01

E1-226 - residues at position E1-226. Exp - experiment number. N m - number of mosquitoes used to estimate OID_50 _value. OID_50 _- oral infectious dose 50%, expressed as Log_10_TCID_50_/mL. FL_95 _- 95% fiducial limits for OID_50 _values expressed as Log_10_TCID_50_/mL. p-value - comparison of statistical significance of difference in OID_50 _values for CHIKV with indicated residues at E1-226 and OID_50 _value of LR-GFP-226A. Comb - combined summary of two independent experiments. NG - data not given.

To further investigate the amino acid requirement at position E1-226 for increased CHIKV midgut infectivity and cholesterol dependence, mutations encoding P, F, M, H and L at E1-226 were introduced into pCHIKV-LR i.c. All constructs had comparable specific infectivity values of *in vitro *transcribed RNAs and produced moderate to high virus titers in BHK-21 cells (Additional file 1, Table 1), indicating that these residues are not lethal for CHIKV, at least in BHK-21 cells. However, constructs with F, M and H residues developed plaques only at 72 hpe in Vero cells, and LR-226L developed plaques at 96 hpe, indicating an attenuated phenotype.

**Figure 3:**
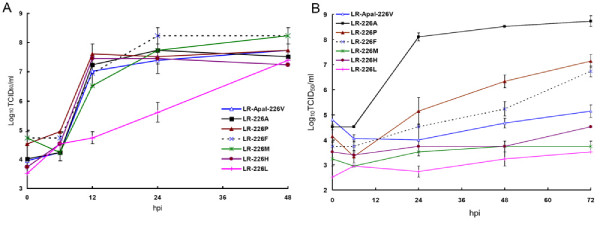
**Effect of P, F, M, H and L residues at E1-226 on growth of CHIKV in standard (A) and cholesterol-depleted (B) C6/36 cells**. Standard cells were infected at an MOI of 1.0. Cholesterol-depleted cells were infected at an MOI of 0.1. Viral titers are expressed as Log_10_TCID_50_/mL ± standard deviation of 2 independent experiments.

**Figure 4:**
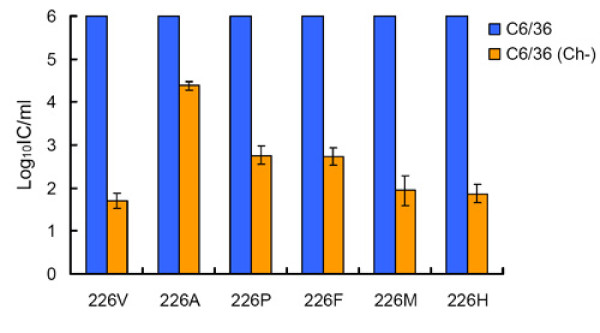
**Effect of P, F, M and H residues at E1-226 on CHIKV infectivity for cholesterol-depleted C6/36 cells**. Standard (blue bars) and cholesterol-depleted (yellow bars) C6/36 cells were infected with serial dilutions of eGFP expressing CHIKV containing indicated residues at E1-226. Results are normalized for 10^6 ^viral infections of standard C6/36 cells. Data indicate an average of three experiments ± standard deviation.

Growth kinetics of all these viruses (except LR-226L) were comparable in standard C6/36 cells (Figure 3A). However, growth of these viruses was significantly inhibited at 24, 48 and 72 hpi in cholesterol-depleted cells as compared to LR-226A or LR226S viruses (Figure 3B) (p < 0.05). Specific infectivity of eGFP-expressing viruses encoding P, F, M, or H at the position E1-226 in cholesterol-depleted C6/36 cells were significantly lower as compared to the infectivity of LR-GFP-226A; however, infectivity of LR-GFP-226P and LR-GFP-226F were significantly higher than that of LR-GFP-226V virus, which agrees with the growth kinetics results (Figure 4). The OID_50 _of LR-GFP-226F for *Ae. albopictus *was similar to the OID_50 _value of LR-GFP-226A (p > 0.1), indicating that E1-226F does not lead to increased CHIKV infectivity (Table 1). Since LR-GFP-226F was found to be much more cholesterol-dependent as compared to LR-GFP-226A, this experiment further supported the earlier observation that dependence for cholesterol does not correlate with increased *Ae. albopictus *midgut infectivity. The OID_50 _values of LR-GFP-226M and LR-GFP-226L were indistinguishable as compared with the LR-GFP-226V and LR-GFP-226I viruses (Table 1), suggesting that V, I, M and L all share similar structural properties important for CHIKV infectivity for *Ae. albopictus*. Interestingly, LR-GFP-226P and LR-GFP-226H demonstrated an intermediate infectivity phenotype, which was statistically different from highly infectious viruses such as LR-GFP-226 [V or I] (p < 0.05) and/or low infectivity viruses such as LR-GFP-226 [A or S] (p < 0.05).

The stability of LR-GFP-226 [I, F, M, H, L] was also analyzed (Additional file 1, Table S3). All mosquitoes infected with CHIKV containing either I, F, M or H residues were found to replicate RNA encoding the I, F, M, and H amino acids at E1-226, respectively, indicating that these viruses remain stable after mosquito infection. Two of four mosquitoes infected with virus derived from the pLR-GFP-226L construct contained RNA encoding only the E1-226L residue. However, sequence results from the remaining two mosquitoes indicated that two RNA species (encoding E1-226L and E1-226P) were simultaneously present in these mosquitoes. These data indicate that LR-GFP-226L is unstable and evolves quickly to eliminate the attenuating E1-226L residue (Additional file 1, Table S3).

### Mutations in other CHIKV genome regions that control cholesterol dependence of CHIKV

The mutagenesis analysis of the position E1-226 in CHIKV showed that all of the residues that confer an increased infectivity phenotype in *Ae. albopictus *(V, I, M and L) also associated with increased dependence for cholesterol in the target membrane. To investigate if increased CHIKV dependence for cholesterol is absolutely required for increased infectivity in *Ae. albopictus*, the LR-ApaI-226V virus, with the E1-226V residue conferring high cholesterol dependence was used to select mutations that can control cholesterol dependence of CHIKV. LR-ApaI-226V was passed four times in cholesterol-depleted C6/36 cells. After the first passage, the virus titer was 5.52 Log_10_TCID_50_/mL at 3 dpi, but at 2 dpi of passage four, the titer was 7.95 Log_10_TCID_50_/mL, indicating successful adaptation to cholesterol-depleted cells. The passage four virus (P4) was plaque purified on Vero cells and 10 individual clones were selected at 2 dpi. Clones were propagated once in C6/36 cells, and the 9500-11000 nt. genome region was sequenced (Additional file 1, Table S4). Seven of 10 sequenced cloned viruses contained the E1-V226A mutation. The second most common variant (3 of 10) contained three nucleotide substitutions (10189 G→T; 10201 G→A; 10660 A→C); encoding two novel mutations (E1-A66S and E1-D70N). In two clones (#1 and #5) these three changes were the only found mutations, but in clone #7, these mutations were accompanied by an E1-V226A substitution. The least common variant (1 out of 10) contained two nucleotide substitutions (10659 G→T and 10970 G→A) leading to two amino acid changes (E1-Q222H and E1-G326D) was excluded form further analysis.

**Figure 5:**
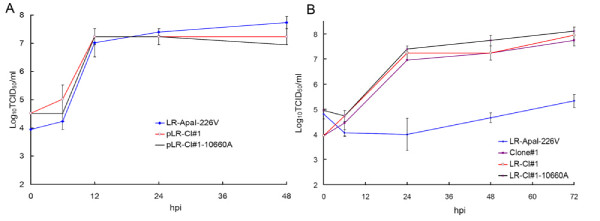
**Growth kinetics of CHIKV with mutations in E1 protein identified in Clone#1 virus in standard (A) and cholesterol-depleted (B) C6/36 cells**. Standard cells were infected at an MOI of 1.0. Cholesterol-depleted cells were infected at an MOI of 0.1. Viral titers are expressed as Log_10_TCID_50_/mL ± standard deviation of 2 independent experiments.

The mutations [10189 G→T(E1-A66S); 10201 G→A(E1-D70N); 10660 A→C (E1-R223R)] from Clone#1 virus were introduced into the pCHIKV-LR i.c. and resultant virus (LR-Cl#1) was tested in standard and cholesterol-depleted C6/36 cells (Figure 5A, B). Growth kinetics of the i.c.-derived virus LR-Cl#1 in cholesterol-depleted C6/36 cells was indistinguishable from cholesterol-adapted virus (Clone#1), and only slightly attenuated as compared to cholesterol-independent virus LR-226A (Figure 5), thereby confirming the role of [E1-A66S and E1-D70N] substitutions in modulating cholesterol dependence of CHIKV. One of these mutations [10660 A→C (E1-R223R)] does not lead to an amino acid substitution; however, it is located in close proximity to position E1-226. This mutation was present in all three individual clones (#1, #5 and #7), suggesting that it might be important for modulating cholesterol dependence of CHIKV (Additional file 1, Table S4). To test this possibility, the point mutation (10660 C→A) from the pLR-ApaI-226V was introduced into pLR-Cl#1, and resultant virus (pLR-Cl#1-10660A) was analyzed in standard and cholesterol-depleted C6/36 cells (Figure 5). This virus replicated as efficiently as LR-Cl#1 or Clone#1 viruses in cholesterol-depleted C6/36 cells indicating that the silent mutation 10660A→C is not implicated in cholesterol sensitivity of CHIKV.

The eGFP-expressing version of LR-Cl#1-10660A (LR-GFP-Cl#1-10660A) was constructed and specific infectivity of resultant virus was determined in standard and cholesterol-depleted C6/36 cells (Additional file 1, Figure S1). The LR-Cl#1-10660A virus was able to infect cholesterol-depleted C6/36 cells significantly more efficiently (p < 0.01 Student's t test) as compared to LR-GFP-226V virus, which genetically corresponds to LR-ApaI-226V virus, used for the adaptation experiment.

To evaluate if the roles of the mutations [E1-A66S and E1-D70N] from Clone #1 virus in modulating cholesterol dependence of CHIKV were correlated with the role of these mutations in CHIKV infectivity to *Ae. albopictus *midguts, the OID_50 _value of LR-GFP-Cl#1-10660A was determined as described above (Table 1). The infectivity of LR-GFP-Cl#1-10660A virus was almost identical to that of LR-GFP-226V (p > 0.1), indicating that [E1-A66S and E1-D70N] mutations do not affect mosquito infectivity of CHIKV. These data also indicate that cholesterol dependence of CHIKV does not directly correlate with increased mosquito infectivity.

### E1-A226V mutation is responsible for decrease in pH threshold for fusion reaction

**Figure 6:**
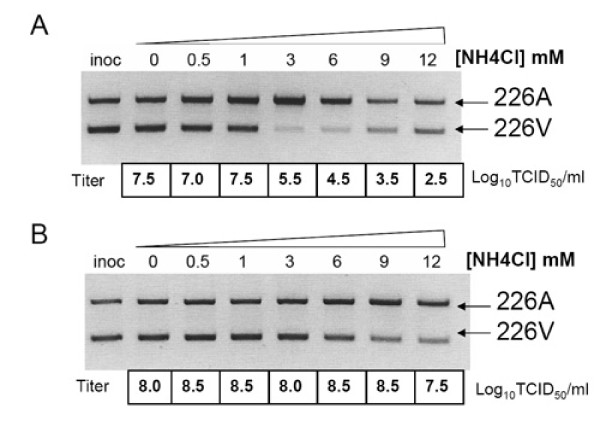
**Effect of NH_4_Cl on competition between LR-ApaI-226V and LR-226A viruses for growth in C6/36 (A) and BHK-21 cells (B)**. Thirty minutes prior to infection cells were pre-incubated with L-15 media containing various amounts of NH_4_Cl. Then cells were infected with 1:1 mixture of LR-ApaI-226V and LR-226A viruses (10^7^pfu/mL) and incubated for 48 h in L-15 media containing various amounts of NH_4_Cl. Cell culture supernatants were harvested and used for RNA extraction or titration on Vero cells. Virus RNA was processed as discussed previously [15].

**Figure 7:**
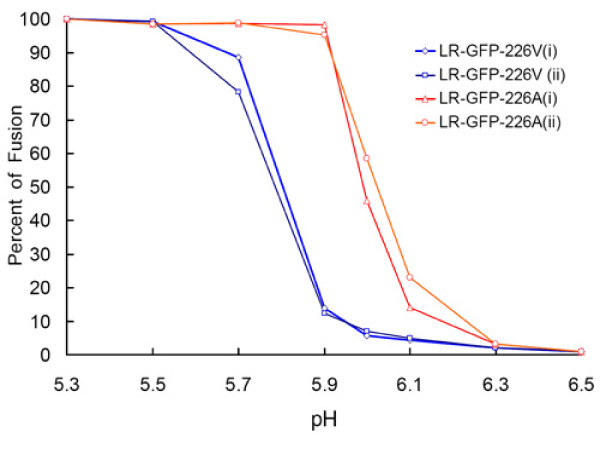
**Effect of E1-A226V mutation on pH dependence of CHIKV induced cell-cell fusion**. C6/36 cells infected with LR-GFP-226A or LR-GFP-226V viruses were incubated for 2 min with L-15 medium whose pH was previously adjusted to desired values. The reaction was abrogated by replacement of the fusion medium with 0.5 mL of standard L-15 medium. Percent of fusion was calculated as (1-c/n)x100%, where c is a number of eGFP expressing cells, n is number of nuclei (n≥70). Two independent experiments were performed for each virus (i and ii). Each individual experiment was performed in duplicates and results are expressed as an average of duplicates.

The pH dependence of the fusion reaction can be analyzed by monitoring the role of agents that act to raise the pH of the endosome in inhibition of viral entry into the cells [34]. The lysotropic agent ammonium chloride (NH_4_Cl) has been used previously to demonstrate the effect of mutations in both SFV and SINV on their fusion thresholds [35,36]. The LR-ApaI-226V and LR-226A viruses were mixed in a 1:1 ratio and used to infect C6/36 or BHK-21 cells pretreated with different concentrations of NH_4_Cl. At 48 hpi cell culture supernatants were collected and either titrated on Vero cells or used for RNA extraction followed by RT-PCR and *Apa*I restrictase digestion to determine the relative RNA ratio of competing viruses. LR-ApaI-226V was markedly more sensitive to inhibition with NH_4_Cl than was LR-226A virus. Thus in C6/36 cells in the presence of 3, 6, and 9 mM of NH_4_Cl, the majority of CHIKV RNA in the supernatant was derived from LR-226A virus (Figure 6A). Similarly, in BHK-21 cells, LR-226A virus outcompeted LR-ApaI-226V at NH_4_Cl concentrations of 9 and 12 mM (Figure 6B). These data suggest that CHIKV with the E1-A226V mutation requires a low pH to trigger the fusion reaction. To confirm these findings, pH-dependence of the cell-cell fusion was compared for LR-GFP-226A and LR-GFP-226V viruses. In two independent experiments the LR-GFP-226V virus triggered cell-cell fusion reaction at ~0.2 lower pH as compared to LR-GFP-226A virus (Figure 7).

**Figure 8:**
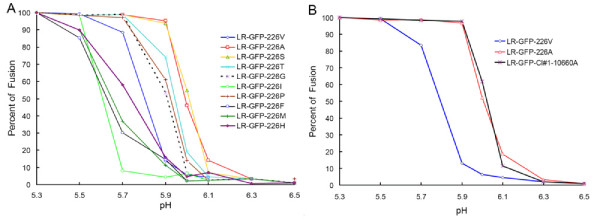
**Effect of mutations at E1-226 (A) and [E1-66/E1-70] (B) on pH dependence of CHIKV induced cell-cell fusion**. C6/36 cells were infected with eGFP expressing viruses and processed as described in legend for Figure 7. Experiments were performed in duplicates for each virus, and results are expressed as an average of duplicates.

To investigate if CHIKV mosquito infectivity phenotypes correlated with different thresholds for their fusion reactions, cell-cell fusion assays were conducted for eGFP-expressing viruses with S, T, G, I, P, F, M and H residues at E1-226 (Figure 8A). No apparent correlation was found between these two parameters. Thus, mosquito infectivity of LR-GFP-226F virus was found to be similar to the infectivity of LR-GFP-226A virus (Table 1), however, LR-GFP-226F virus triggered the cell-cell fusion reaction at a pH even lower than the pH of fusion of the highly infectious virus LR-GFP-226V (pH~5.6 vs pH~5.8 respectively). Interestingly, mosquito infectivity of LR-GFP-Cl#1-10660A virus was identical to that of LR-GFP-226V (Table 1), however, LR-GFP-Cl#1-10660A triggered fusion reaction in C6/36 cells at pH markedly higher than LR-GFP-226V (Figure 8B).

## Discussion

The detailed mutagenesis analysis of position E1-226 demonstrated that CHIKV can tolerate a significant variability of amino acids at position E1-226. Depending on specific residues, CHIKV exhibited various phenotypes in cell culture conditions. These phenotypes may be broadly categorized as follows (Additional file 1, Table S1, S2): 1) - viruses that have S, T, G, P were almost indistinguishable from parental (wild-type) virus containing V or A; 2) - introduction of I, F, M or H residues resulted in viruses that were slightly attenuated; 3) - introduction of L residues resulted in significant attenuation of CHIKV. Analysis of viruses with specific mutations at E1-226 in *Ae. albopictus *mosquitoes revealed that effects of these mutations can also be classified into three groups as follows: 1) - amino acids I, M and L resulted in viruses that were highly infectious to midgut cells of *Ae. albopictus*. The OID_50 _values of these viruses were not significantly different from CHIKV with E1-226V; 2). Viruses with amino acids S, T, G or F at E1-226 were associated with a low infectivity phenotype for *Ae. albopictus*. The OID_50 _values of these viruses were statistically indistinguishable from the ancestral form of CHIKV with E1-226A residue. 3) - an intermediate infectivity phenotype was attributed to P and H residues. OID_50 _values of these viruses were statistically higher than that of highly infectious viruses (V, I, M and L), but in the same time lower than that of low infectivity viruses (Table 1).

To investigate the effect of mutations at E1-226 on the CHIKV requirement for cholesterol, growth kinetics and specific infectivity were compared for CHIKV with different mutations at E1-226 in cholesterol-depleted C6/36 cells. The pre-epidemic CHIKV (E1-226A) infected and replicated much more efficiently in cholesterol-depleted C6/36 cells as compared to the mutated CHIKV (V, I, M, or L at E1-226) with a high infectivity phenotype in *Ae. albopictus *(Figure 1, 2, 3, 4). This observation suggests that CHIKV dependence for cholesterol might be associated with infectivity to *Ae. albopictus*. Interestingly, a recent study showed that the E1 protein of alphaviruses can directly bind to cholesterol, which influences fusogenic properties of the viruses [37]. This suggests that direct E1 binding to cholesterol can be responsible for an increase in CHIKV infectivity for *Ae. albopictus*. However, analysis of viruses that showed intermediate and decreased infectivity to *Ae. albopictus *does not support the conclusion that CHIKV dependence for cholesterol is mechanistically correlated with infectivity to *Ae. albopictus*. These viruses demonstrated a broad spectrum of cholesterol dependence phenotypes that do not correlate with mosquito infectivity profile. The simplest explanation for the lack of correlation is that dependence for cholesterol and infectivity to *Ae. albopictus *are two independent phenotypic effects of the E1-226 mutations. Alternatively, dependence for cholesterol might be controlled at several steps of the infectious process. Cholesterol-independent viruses (such as LR-226A and LR-226S) do not rely on cholesterol at each of these steps. However, cholesterol-dependent viruses (such as LR-226V and LR-226G) may require cholesterol at different (not overlapping) steps, and the CHIKV-cholesterol interaction at steps which are connected to *Ae. albopictus *infectivity are unnecessary for viruses that showed decreased infectivity for *Ae. albopictus*.

All of the residues at E1-226 that are associated with high infectivity for *Ae. albopictus *also demonstrated high dependence to cholesterol. To address the question if cholesterol dependence is absolutely required for high mosquito infectivity, we identified mutations in the CHIKV genome regions other than E1-226, which can make CHIKV less cholesterol-dependent (Figure 5, Additional file 1, Table S4). Introduction of E1-A66S and E1-D70N mutations into the i.c. of CHIKV with valine at E1-226 significantly releases cholesterol dependence of CHIKV; however, these mutations had no effect on CHIKV infectivity to *Ae. albopictus *mosquitoes. These data indicate that cholesterol dependence can be modulated independently of the phenotypes of infectivity to *Ae. albopictus *mosquitoes. Altogether, these observations and previously discussed data on the roles of individual mutations at E1-226 indicate that cholesterol dependence of CHIKV cannot serve as an indicator of infectious properties of CHIKV in *Ae. albopictus*.

Interestingly, all substitutions that control cholesterol dependence of CHIKV occurred in an E1 region that has never previously been implicated in modulating cholesterol dependence of alphaviruses. Previously it was shown that besides the position E1-226, the mutations in the "hinge" region of the E1 protein (positions: E1-44 and E1-178) could also control cholesterol dependence of SFV. In addition, E1-44 and E1-178 mutations control SFV dependency for sphingolipids, and it was proposed that these mutations in the hinge region control relative flexibility of domain I and domain II of E1, which are believed to be important for the biological function of the molecule [38]. The effector mechanism of mutations from Clone#1 (E1-A66S and E1-D70N) and Clone#10 [(Q222H) data not shown] on CHIKV cholesterol dependence are unknown. The mutations E1-A66S and E1-D70N are located in the bc loop of domain II which is involved in interactions with Domain I of the adjacent E1 protein and E2 protein [17,25]. Therefore, it is possible that these mutations generally affect the stability of the CHIKV particle. The Q222H is located in the middle of the ij loop of E1 and probably acts by controlling the flexibility and the topology of the tip of this loop, which interacts with the fusion loop and target membrane.

The common feature shared by residues at E1-226 that ensure a high infectivity phenotype of CHIKV in *Ae. albopictus *(V, I, L and M) is that they belong to class of non-polar aliphatic amino acids. Interestingly, all residues that do not fall into this category (A, S, T, G, F, P, H) were associated with significantly lower infectivity to *Ae. albopictus*. It appears that a general requirement for an increase in infectivity for *Ae. albopictus *is associated with the presence of at least two or more aliphatic carbon atoms connected to the β-carbon of alanine. The current model of the alphavirus membrane fusion process postulates that upon exposure to low pH and E2-E1 heterodimer dissociation, the fusion loop and/or adjacent ij loop (with position E1-226) act as "sensor of lipid composition" of target membranes, and then regulate the stable insertion of the fusion loop into membrane of endosomes [26,39,40]. Therefore, the aliphatic groups of V, I, M and L residues at the position E1-226 of ij loop could be directly involved in modulation of this "lipid sensor" step via direct interaction with target membrane or by changing conformation of the fusion loop, which is located in close proximity to the ij loop. However, we also cannot exclude the possibility that mutations at the tip of the ij loop (E1-226) act independently of the fusion loop by changing overall conformation of E1 protein during this "lipid sensing" step.

Competition experiments between LR-ApaI-226V and LR-226A in the presence of inhibitor of endosomal acidification (NH_4_Cl), and cell-cell fusion experiments demonstrated that the E1-A226V mutation is also responsible for a decrease in the pH required for triggering of a fusion reaction (Figure 6, 7). The observed difference in pH dependence between LR-GFP-226V and LR-GFP-226A viruses was relatively small (~0.2); however, it is possible that it can have significant impact on the location of where the fusion reaction occurs within endosomal compartments. Thus, lower pH suggests that fusion of E1-226V viruses would occur not in early endosomes (pH 6.5-6.0), but rather in late endosomes (pH < 6.0), which have quite different lipid composition [41-44]. Interestingly, residues E1-226I, and E1-226M that were found to significantly increase CHIKV infectivity to *Ae. albopictus*, also led to a decrease in the pH of fusion reaction. However, the further analyses of the effects of different mutations at E1-226 on pH of fusion reaction failed to support the association between pH-dependence of the fusion reaction and CHIKV phenotypes in *Ae. albopictus *(Figure 8). This indicates that a decrease in the pH of fusion reaction due to E1-A226V mutation is probably not directly responsible for the observed increase in CHIKV infectivity to *Ae. albopictus *mosquitoes.

There was also no clear correlation between pH and cholesterol dependence of CHIKV. Thus, although it seems that viruses that have lower cholesterol dependence for cell entry have a tendency to trigger fusion at higher pH, there are two exceptions that do not fit this relationship. The E1-226F mutation was responsible for intermediate dependency for cholesterol, but required very low pH to trigger fusion. However, CHIKV with E1-226G was highly cholesterol-dependent, but triggered fusion at markedly higher pH than the other highly cholesterol-dependent virus. Interestingly, the E1-P226S mutation in SFV which is also responsible for modulation of cholesterol dependence, has no effect on SFV pH dependence for cell entry [39].

It remains perplexing why wild-type isolates of some alphaviruses such a SFV and SINV are highly dependent on the presence of cholesterol in membranes of host cell [21,23], whereas others such as CHIKV [15] and VEEV [27] can resist cholesterol depletion? It is particularly surprising considering the fact that biological transmission of alphaviruses occurs in mosquito vectors that do not have enzymatic pathway to synthesize cholesterol and obtain required sterols directly from the diet [45,46]. Our data shows that dependence for cholesterol is not directly associated with mosquito infectivity or pH dependence phenotypes. Moreover, it was shown that a cholesterol-independent mutation E1-P226S in SFV confers more efficient viral replication in intrathoracicaly injected *Ae. albopictus *compared to highly cholesterol-dependent wild type SFV [47], however, selection of this mutation by SFV strains in nature has never been documented. Since mutations that control cholesterol dependence also control many other unrelated functions of the alphaviruses, we hypothesize that cholesterol dependence/independence is not associated with any specific evolutionary force that determines alphavirus evolution in nature, and is acquired/lost by particular viruses as a result of viral adaptation to other conditions/functions associated with specific host specie used by viruses in given ecological settings.

## Conclusions

In summary, we demonstrated that mutation E1-A226V is associated with multiple effects on CHIKV physiology, such as cholesterol and pH dependency and mosquito infectivity. The detailed analyses of CHIKV with different mutations at E1-226 and other genome regions revealed that there is no clear correlation between these parameters. This indicates that the E1-A226V mutation probably acts at different steps of CHIKV life cycle affecting multiple functions of the virus.

## Authors' contributions

KAT designed the experiments; KAT, CEM performed experiments, KAT, CEM and SH analyzed the data and wrote the paper. All authors read and approved the final manuscript.

## Competing interests

The authors declare that they have no competing interests.

## Supplementary Material

Additional file 1 **Figure S1, Table S1, Table S2, Table S3 and Table S4**. The file contains Figure S1, Table S1, Table S2, Table S3, Table S4 and legends for these Figure and Tables. Click here for file
