# Supplementary material for: Chikungunya virus adaptation to Aedes albopictus mosquitoes does not correlate with acquisition of cholesterol dependence or decreased pH threshold for fusion reaction
Source: Virol J. 2011 Jul 29;8:376. doi: 10.1186/1743-422X-8-376 (PMC3162544; doi:10.1186/1743-422X-8-376)
Supplement: Additional file 1 — Figure S1, Table S1, Table S2, Table S3 and Table S4. The file contains Figure S1, Table S1, Table S2, Table S3, Table S4 and legends for these Figure and Tables. [file 1743-422X-8-376-S1.PDF]

Additional file 1:

Figures and Tables legends:

Figure S1. Effect of [E1-A66S and E1-D70N] mutations on CHIKV infectivity for cholesterol-depleted C6/36 cells.

Standard (blue bars) and cholesterol-depleted (yellow bars) C6/36 cells were infected with serial dilutions of LR-GFP-Cl#1-10660A. Results are normalized for 10^6 viral infections of standard C6/36 cells. Data indicate an average of three experiments ± standard deviation.

Table S1. Specific infectivity of *in vitro* transcribed RNA and titers after RNA electroporation for non-GFP expressing viruses with substitutions at E1-226.

a – amino acids at position E1-226.

b – time (h) required for visible plaques to develop.

c – Virus titers were determined by titration on Vero cells and expressed as $Log_{10}TCID_{50}$/mL.

ND- titer not determined.

Table S2. Specific infectivity of *in vitro* transcribed RNA and titers after RNA electroporation for GFP-expressing viruses with substitutions at E1-226.

a – amino acids at position E1-226.

b – time (h) required for visible plaques to develop.

c – Virus titers were determined by titration on Vero cells and expressed as $Log_{10}TCID_{50}$/mL.

ND- titer not determined.

Table S3. Stability of selected mutations at the position E1-226 in *Ae. albopictus* mosquitoes.

Time - time (h) required for visible plaques to develop.

N m - mumber of individual mosquitoes analyzed.

E1-226 – residue at E1-226 determined by sequencing of CHIKV RNA isolated from individual mosquito.

P/L means that CHIKV with leucine and proline ar E1-226 are simultaneously present in this mosquito.

Table S4. Summary of adaptation experiment.

a – identified nucleotide mutations and genome location for each of plaque purified viruses.

b – corresponding amino acid substitutions.

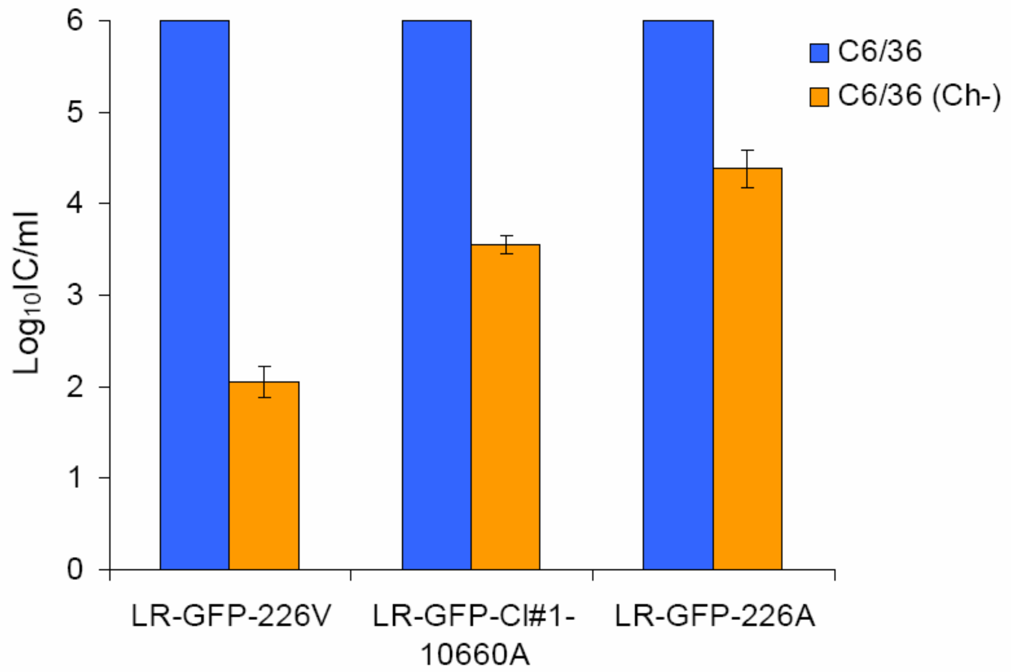

Figure S1. Effect of [E1-A66S and E1-D70N] mutations on CHIKV infectivity for cholesterol-depleted C6/36 cells.

Table S1. Specific infectivity of *in vitro* transcribed RNA and titers after RNA electroporation for non-GFP expressing viruses with substitutions at E1-226.

Template used for *in vitro* transcription.	E1-226 [a]	Time [b]	Specific infectivity (pfu/µg of RNA)	Virus titer[c]	
				24 h	48 h
pLR-ApaI-226V	V	48	9.1×10^5	6.95	7.52
pLR-226A	A	48	8.3×10^5	6.52	7.52
pLR-226S	S	48	7.0×10^5	7.95	7.95
pLR-226T	T	48	6.5×10^5	7.95	7.95
pLR-226G	G	48	10.0×10^5	6.52	7.52
pLR-226I	I	72	5.0×10^5	6.52	6.52
pLR-226P	P	48	9.5×10^5	7.52	6.52
pLR-226F	F	72	9.8×10^5	6.95	6.52
pLR-226M	M	72	9.9×10^5	7.52	7.52
pLR-226H	H	72	10.0×10^5	6.95	6.95
pLR-226L	L	96	10.0×10^5	6.95	6.95

Table S2. Specific infectivity of *in vitro* transcribed RNA and titers after RNA electroporation for GFP-expressing viruses with substitutions at E1-226.

Template used for *in vitro* transcription.	E1-226 [a]	Time [b]	Specific infectivity	Virus titer[c]	
				24h	48h
pLR-GFP-226V	V	48	$4.8x10^5$	6.52	6.52
pLR-GFP-226A	A	48	$3.3x10^5$	6.95	6.95
pLR-GFP-226S	S	48	$7.0x10^5$	7.95	7.95
pLR-GFP-226T	T	48	$6.5x10^5$	7.95	7.95
pLR-GFP-226G	G	48	$10.0x10^5$	6.52	7.52
pLR-GFP-226I	I	72	$5.0x10^5$	6.52	6.95
pLR-GFP-226P	P	48	$7.2x10^5$	6.95	6.95
pLR-GFP-226F	F	72	$5.0x10^5$	ND	6.95
pLR-GFP-226M	M	72	$5.0x10^5$	ND	6.72
pLR-GFP-226H	H	72	$5.0x10^5$	5.95	6.95
pLR-GFP-226L	L	96	$3.0x10^5$	5.95	6.52

Table S3. Stability of selected mutations at the position E1-226 in *Ae. albopictus* mosquitoes.

Virus	Time	N m	E1-226
LR-GFP-226I	72	4	1-I; 2-I; 3-I; 4-I
LR-GFP-226F	72	3	1-F; 2-F; 3-F
LR-GFP-226M	72	3	1-M; 2-M; 3-M
LR-GFP-226H	72	3	1-H; 2-H; 3-H
LR-GFP-226L	96	4	1-L; 2-L/P; 3-L/P, 4-L

Table S4. Summary of adaptation experiment.

Clone #	Mutation (nt)[a]	Mutation (aa)[b]
1	10189 G→T<br>10201 G→A<br>10660 A→C	E1-A66S<br>E1-D70N<br>E1-R223R
2	10760 T→C	E1-V226A
3	10760 T→C<br>10337 A→C	E1-V226A<br>E1-K114T
4	10760 T→C	E1-V226A
5	10189 G→T<br>10201 G→A<br>10660 A→C	E1-A66S<br>E1-D70N<br>E1-R223R
6	10760 T→C	E1-V226A
7	10189 G→T<br>10201 G→A<br>10660 A→C<br>10760 T→C	E1-A66S<br>E1-D70N<br>E1-R223R<br>E1-V226A
8	10760 T→C	E1-V226A
9	10760 T→C	E1-V226A
10	10659 G→T<br>10970 G→A	E1-Q222H<br>E1-G326D
